# Isolation and Identification of Flavanone Derivative Eriodictyol from the Methanol Extract of *Afzelia africana* Bark and Its Antimicrobial and Antioxidant Activities

**DOI:** 10.1155/2023/9345047

**Published:** 2023-05-09

**Authors:** Bright Yaw Vigbedor, Clement Osei Akoto, David Neglo

**Affiliations:** ^1^Department of Basic Sciences, School of Basic and Biomedical Sciences, University of Health and Allied Sciences, Ho, Ghana; ^2^Department of Chemistry, Faculty of Physical and Computational Sciences, College of Science, Kwame Nkrumah University of Science and Technology (KNUST), Kumasi, Ghana

## Abstract

**Background:**

*Afzelia africana* is a plant species with well-documented ethnobotanical and medicinal properties. The plant is reported to have various secondary metabolites and had been applied for the treatment of various diseased conditions.

**Objectives:**

The study objectives include fractionation, isolation, purification, and characterization of eriodictyol from the bark of *A. africana*, and the determination of its antimicrobial and antioxidant activities. *Methodology*. The series of methodologies that were employed include fractionations and purification (column chromatography), characterization (HPLC, LC-MS, IR, ^1^H, ^13^C, DEPT-135, HSQC, and HMBC), antimicrobial assays (microbroth dilution and checkerboard assay), and antioxidant activities assays (ABTS and DPPH scavenging capacity).

**Results:**

The study reports the identification and characterization of eriodictyol from the bark of *A. africana* which exhibited potent antioxidant activities against ABTS and DPPH radicals with scavenging capacities (SC_50_) of 2.14 ± 0.05 and 2.51 ± 0.06 *µ*g/mL, respectively. The compound exhibited its antimicrobial activity by reporting good bacteriostatic activities (MBC/MIC > 4) against *Staphylococcus aureus* (SA), methicillin-resistant *Staphylococcus aureus* (MRSA), and fluconazole-resistant *Candida albicans* (CA2). Moreover, a broad spectrum of bactericidal effects (MBC/MIC ≤ 4) was reported against *Streptococcus mutans* (SM), *Escherichia coli* (EC), *Bacillus subtilis* (BS), *Klebsiella pneumonia* (KP), *Pseudomonas aeruginosa* (PA), *Salmonella typhi* (ST), and standard *Candida albicans* (CA1). The compound further exhibited synergistic effects against EC, KP, ST, and MRSA; ST; and CA2 when combined with ciprofloxacin, tetracycline, and nystatin, respectively. However, antagonistic effects were observed against PA and CA1 when combined with ciprofloxacin and ketoconazole, respectively.

**Conclusion:**

The study reports for the first time the identification of eriodictyol from the bark of *A. africana* which exhibited significant antioxidant and antimicrobial properties.

## 1. Introduction

The statistical data from the World Health Organization suggest that 80% of developing countries depend on plant medicines for the treatment of diseases and ailments through ethnobotany. As a result, the ethnobotanical study has provided a rich source of medicinal information from plants [1]. The information has contributed to the incorporation of herbal medicine into the main health systems of various national health care systems [1, 2]. Research beyond the ethnobotanical information has contributed to a deeper understanding of the phytochemistry, pharmacognosy, and ethnopharmacology of herbal medicines [3, 4]. Consequently, strategies for the fractionation and purification of plant extracts based on biological activity have led to the isolation of bioactive compounds with subsequent application as drugs or lead agents for further drug development protocols [3, 5, 6].

Africa is endowed with a vast floral scope that has been utilized ethnobotanically for the treatment of diseases. One plant with enormous ethnomedicinal utilization is *Afzelia africana*, a plant species belonging to the Fabaceae family [7]. The plant is commonly referred to as African mahogany with medium to large sizes and can grow up to 40 m [8]. The medicinal prowess of *A. africana* has been reported through various ethnobotanical surveys. The plant has been utilized ethnobotanically as an analgesic, anti-inflammatory, antidiabetic, antimalarial, aphrodisiac, antimicrobial, emetic, emmenagogic, trypanocidal, and laxative agents [7, 9]. The antitrypanocidal, anthelmintic, antidiabetic, antimicrobial, and synergistic effects on standard antibacterial and fungal agents were also well-reported [10–14]. Moreover, the methanol extract of the plant was reported to have higher susceptibility against bacterial isolates such as *Escherichia coli*, *Staphylococcus aureus*, and *Candida albicans* [15, 16]. Investigation of the phytochemical constituents of the plant extract revealed the presence of flavonoids, tannins, saponins, alkaloids, and steroids [9, 13]. Furthermore, the ethnobotanical usage of the plant as an antiplasmodial agent was authenticated against the 3D7 strain of *Plasmodium falciparum* [9, 14]. Despite its rich medicinal potential, little or no compound has been isolated and linked to its numerous biological activities even though the plant is endowed with rich phytochemicals. A particular phytochemical class consistently identified in *A. africana* is a flavonoid. Flavonoid compounds are further classified into several derivatives. Particularly, flavanones are flavonoid derivatives with the basic 2,3-dihydroflavone structure with reported numerous health benefits. Unlike their other flavonoids counterpart, they lack a double bond at the C2-C3 portion of their chromene-4-one moiety [17]. They are biosynthesized and abundant in citrus fruits. The main aglycone naringenin, hesperetin, and eriodictyol are, respectively, abundant in grapefruit, oranges, and lemons [18]. They are also abundantly available in various parts of cereals and other plant families such as Compositae, Leguminosae, and Rutaceae [19]. The study sought to employ the principle of bioassay-guided fractionation studies to isolate, purify, and characterize a flavanone compound from the bark of *A. africana* and to evaluate its antimicrobial and antioxidant activities.

## 2. Materials and Methods

### 2.1. Plant Materials

The bark of *A. africana* was collected from the forest of Nsuta (7.0129° N, 1.3783° W) in the Sekyere Central District of the Ashanti region of Ghana. The plant was authenticated by Mr. Clifford Osafo Asare, and a voucher specimen sample (specimen number: KNUST/HM1/2019/SB/008) was deposited in the Herbarium unit of the Department of Herbal Medicine, Faculty of Pharmacy and Pharmaceutical Sciences, KNUST, Ghana.

### 2.2. Chemicals and Reagents

All chemicals were purchased from Sigma Aldrich Co. Ltd, Irvine, UK, except the standard drugs. The chemicals were methanol (analytical grade), ethyl acetate (analytical grade), petroleum ether (analytical grade), chloroform (analytical grade), hexane (analytical grade), DPPH (analytical grade), ABTS (analytical grade, Korea), DMSO (analytical grade), and silica gel 60 (230–400 mesh, analytical grade).

### 2.3. Extraction of Plant Material

The bark of the plant material was air-dried under shade and pulverized. 500 g of the pulverized plant material was macerated in 90% methanol for three days with periodic agitation, followed by filtration. The filtered extract was then concentrated and evaporated to dryness using the rotary evaporator (BUCHI Rotavapor R-114) at 50°C. The percentage yield of the extract was determined to be 12% w/w (60 g) and kept at 2°C until further use.

### 2.4. Column Chromatographic Fractionation

The dried crude extract (60 g) was subjected to a flash column chromatography (45 × 4.5 cm) containing 500 g silica gel 60 (mesh size: 70–230) as previously described [20]. The extract was fractionated by eluting with solvent compositions comprising petroleum ether, petroleum ether-ethyl acetate (PE-EA), and ethyl acetate as the mobile phase. The resulting column chromatography fractions based on their thin-layer chromatography (TLC) profile yielded fractions F1 (PE-EA (85 : 15), 200 mg), F2 (PE-EA (75 : 25), 342 mg), and F3 (PE-EA (70 : 30), 300 mg). The fraction F3 was further fractionated on 50 g silica gel 60 (mesh size: 70–230), using PE-EA (70 : 30) to afford a 20 mg yellow crystal. The compound was further recrystallized in hot ethanol. The characterization and biological activities were determined.

### 2.5. Structural Elucidation

The structure of the isolated compound was characterized by employing analytical techniques such as HPLC, FTIR, LC-MS, MS, and NMR.

#### 2.5.1. HPLC Condition for Purity Determination

The HPLC chromatogram was determined on the PerkinElmer (PE) series 200 (Kyoto, Japan) liquid chromatography using the BDS Hypersil C18 reversed-phase column (250 × 4.6 mm, 5 *μ*m). The isolated compound was solubilized in 5.0 mL of methanol and 20 *μ*L injected into the HPLC using methanol-water-formic acid (40 : 60 : 1) as the mobile phase. The compound was detected at a flow rate and wavelength of 1 mL/min and 254 nm, respectively, using the diode array UV detector.

#### 2.5.2. FTIR Spectroscopy

The IR spectrum of the compound was determined using the FTIR (820IA single beam laser Shimadzu infrared spectrophotometer, (Tokyo, Japan)) within the wavenumber regions of 4,000 cm^−1^– 400 cm^−1^.

#### 2.5.3. LC-MS

The LC-MS of the compound was determined using the LC system (Terkinelmer Agilent infinity 1,290 LC coupled to the 6,420 triple quadrupole MS (Melbourne, Australia)). The solvent system consisted of methanol-water (50 : 50) at a flow rate of 1.5 mL/min.

#### 2.5.4. ESI-MS

The ESI-MS (positive ion mode) was obtained on an LC system coupled to an Agilent single quadrupole mass-selective detector (Terkinelmer Agilent infinity 1,290 LC, 6,420 triple quadrupole MS (Melbourne, Australia)). The MS was generated at the following conditions: fragmentor voltage = 100 V, capillary voltage = 2500 V, nebulizer pressure = 30 psi, drying gas temperature = 350°C, and mass range = 100–600 Da. The fragmented ions were separated according to their mass to charge ratios (m/z).

#### 2.5.5. NMR Spectroscopy

The ^1^H and ^13^C NMR spectra of the compound were determined at 500 and 125 MHz, respectively, using the Bruker 500 MHz NMR facility. The 2-D spectra that were also performed included DEPT-135, HSQC, and HMBC.

#### 2.5.6. Melting Point

The melting point of the compound was determined on the meting point apparatus (R000105350; Stuart, UK).

#### 2.5.7. Optical Rotation

The optical rotation of the compound (0.02 g/mL in ethanol) was determined using the BS Bellingham/+Stanley (Kent, UK) polarimeter (sample tube: 100 mm; light source: sodium lamp (D, 589 nm); temperature: 20°C).

### 2.6. Antioxidant Activity

Two main assays, the ABTS and DPPH free radicals scavenging activity, were used for the determination of the antioxidant activity of the plant compound.

#### 2.6.1. ABTS Radical Scavenging Activity

The ABTS free radical scavenging capacity of the compound was examined with reference to a previous procedure by Re et al. and Sebastian et al. [21, 22] with minor optimization. The activity was performed by mixing the ABTS (10 mL) and 2.4 mM potassium persulphate (10 mL) to produce the ABTS free radical. The resulting solution was diluted with 50 mL of methanol to generate the working stock solution. A 0.150 mL of the stock solution was mixed with 0.050 mL of the prepared compound with concentrations (C) of 100.00, 50.00, 25.00, 12.50, 6.25, and 3.13 *µ*g/mL. The resulting mixtures were vortexed and incubated at 30°C for 30 min. The absorbances of the mixtures were measured with the UV spectrophotometer (Jenway, Bibby Scientific Ltd, Staff, UK) at 734 nm for control ABTS (*Ao*) and the test samples (*A*). The experimental protocol was repeated for ascorbic acid with concentrations of 100.00, 50.00, 25.00, 12.50, 6.25, and 3.13 *µ*g/mL. The experimental procedure was performed in triplicate. The percentage (%) scavenging activity was evaluated by employing the relation as follows:

(1) $$\begin{array}{c} \%\;ABTS\;scavenging\;activity=\left[\frac{\left(Ao\right)-\left(A\right)}{\left(Ao\right)}\right]\times 100\text{.} \end{array}$$

A graph of the % scavenging activity against the logarithm of the concentration [Log *C*] was plotted. The fifty percent scavenging capacity ((SC_50_), *µ*g/mL) was deduced from the regression equation [*Y* = *mx* + *C*] from the graphs by evaluating the relation at *Y* = 50:

(2) $$\begin{array}{c} {SC}_{50}\;\left(\frac{µg}{mL}\right)=\log^{-1}\left[\frac{50-C}{m}\right]\text{,} \end{array}$$

where *m* and *C* are the slope and intercept from the regression equation.

#### 2.6.2. DPPH Radical Scavenging Activity

The DPPH scavenging activity of the compound was determined with reference to the method described by Vigbedor et al. and Osei Akoto et al. [20, 23] with minor optimization. 0.1 mM stock solution of DPPH was prepared by dissolving 394 *µ*g in 10 mL of ethanol. 0.5 mL of the solution was added to 1 mL of the compound with concentrations of 100.00, 50.00, 25.00, 12.50, 6.25, and 3.13 *µ*g/mL. The resulting solutions were vortexed and incubated at 30°C for 30 min. The absorbances of the solutions were recorded at 517 nm. The procedure was repeated for the reference standard (ascorbic acid) with concentrations of 100.00, 50.00, 25.00, 12.50, 6.25, and 3.13 *µ*g/mL. The experimental procedure was performed in triplicate. The percentage (%) scavenging activity of the compound was calculated by inputting the absorbance values into the relation with *A* and *Ao* being the absorbances of the compound and DPPH, respectively:

(3) $$\begin{array}{c} \%\;DPPH\;scavenging\;activity=\left[\frac{\left(Ao\right)-\left(A\right)}{\left(Ao\right)}\right]\times 100\text{.} \end{array}$$

A plot of the % scavenging activity versus the logarithm of [Log *C*] was performed and the SC_50_ (*µ*g/mL) was deduced from the regression equation [*Y* = *mx* + *C*] from the graphs by evaluating the relation at *Y* = 50:

(4) $$\begin{array}{c} {SC}_{50}\;\left(\frac{µg}{mL}\right)=\log^{-1}\left[\frac{50-C}{m}\right]\text{,} \end{array}$$

where *m* and *C* are the slope and intercept from the regression equation.

### 2.7. Antimicrobial Activity

The antimicrobial activity of the isolated compound was accessed by evaluating the minimum inhibitory concentration (MIC), minimum bactericidal concentration (MBC), and minimum fungicidal concentration (MFC) by employing the micro broth dilution assay. The synergistic effect was determined by evaluating the fractional inhibitory concentration (FIC) index using the checkerboard assay.

#### 2.7.1. Sources of Microorganisms

The bacteria and fungi strains were obtained from the Microbiology Unit of the School of Basic and Biomedical Sciences, University of Health and Allied Science (UHAS), Ho. The microbial strains that were investigated include *Bacillus subtilis* (ATCC 10004), *Candida albicans* 1 (ATCC 90028), fluconazole-resistant *Candida albicans* 2 (clinical strain), *Escherichia coli* (NCTC), *Klebsiella pneumonia* (NCTC 13440), 12241), methicillin-resistant* Staphylococcus aureus* (NCTC 12493), *Staphylococcus aureus* (NCTC 12973), *Pseudomonas aeruginosa* (ATCC), *Streptococcus mutans* (ATCC 700610), 4853), and *Salmonella typhi* (ATCC 14028).

#### 2.7.2. Inoculum Preparation

The microbial strains were prepared by growing them on the nutrient agar (Oxoid, United Kingdom) plates for 24 h at 37°C in an incubator. The direct colony suspension method was employed to transfer colony of suspensions from the plates into 10 mL of sterile saline in test tubes using a sterile inoculating loop. The turbidities of the colonies were adjusted and compared to the 0.5 McFarland standards. The absorbances were recorded at 625 nm using the UV/visible spectrophotometer (Jenway, Bibby Scientific Ltd, Staff, UK). The inoculums were finally diluted to 10^3^ (colony-forming unit) CFU/mL [23].

#### 2.7.3. Minimum Inhibitory Concentration (MIC)

The MIC of the compound was evaluated by employing the micro broth dilution method 96-well plate (Citotest Labware Manufacturing Co. Ltd, Jiangsu, China) according to the Clinical and Laboratory Standards Institute (CLSI) guidelines and [23–26] with minor modifications. 10 mg/mL stock solution was prepared by weighing 10 mg and then dissolving in 1 mL of DMSO. A two-fold serial dilution was prepared until 10 different concentrations were obtained. An aliquot of 100 *μ*L of double-strength Mueller Hinton broth (Oxoid Limited, United Kingdom) was dispensed into each 96-well plate and mixed with 100 uL of the compound to prepare well concentrations ranging from 100.0 to 0.1 mg/mL. Specific wells served as both positive (broth + organism only) and negative (broth with no organism) controls, respectively, for each microorganism on each column. This process was repeated for the antibiotics, ciprofloxacin, tetracycline, fluconazole, nystatin, and ketoconazole at concentrations range of 256.0−0.125 *µ*g/mL. The procedure was followed by the addition of 100 *µ*L of each of the 0.5 McFarland standardized and test organisms. The plates were incubated at 37°C for 48 h. The 3-(4,5-dimethylthiazol-2-yl)-2,5-diphenyltetrazolium bromide (MTT) dye was added to each well and then incubated for about 30 min. The MIC values were then evaluated by visual analysis.

#### 2.7.4. Minimum Bactericidal (MBC) and Fungicidal Concentration (MFC)

To confirm if the isolated compound would be able to kill the microbial cells (bactericidal/fungicidal effect), the MBC and MFC were determined. Aliquots from each well from susceptibility testing assays were transferred to plates containing nutrient agar and then incubated for 48 h at 37°C. The plates were then checked for the presence or absence of growth in the nutrient agar or Sabouraud dextrose agar (SDA) [26].

#### 2.7.5. Determination of the Synergistic Effect of the Isolate with Selected Antimicrobial Agents

*In vitro* analysis of the interaction between the isolate and the antibiotics (tetracycline and ciprofloxacin) against the bacterial strains was evaluated by modifying the procedure previously reported by Khodavandi et al. and Dickson et al. [27, 28]. The test concentrations for each antibiotic and the isolate used range from 1/32MIC to 2xMIC. The experimental procedure was further repeated for the fungal isolate (*C. albicans*) with antifungal agents such as fluconazole, ketoconazole, and nystatin. The synergistic effects were determined by computing the fractional inhibitory concentration (FIC) index from the relation:

(5) $$\begin{array}{c} FIC\;index=\frac{\left[MIC\;of\;antibiotic\;in\;combination\right]}{\left[MIC\;of\;antibiotic\;alone\right]}+\frac{\left[MIC\;of\;compound\;in\;combination\right]}{\left[MIC\;of\;compound\;alone\right]}\text{.} \end{array}$$

The interaction was considered synergistic if the FIC index was ≤0.5; partial synergistic if the FIC index was >0.5 and <1; additive if FIC index was = 1; indifference if the FIC index was >1 and ≤4; and antagonistic if the FIC Index was >4.0.

### 2.8. Statistical Analysis of Data

All the data generated from the experiments were analyzed with the GraphPad Prism software package 5.00 for Windows (San Diego, California, USA). The data were further subjected to one-way ANOVA analysis (*P* < 0.01). The antioxidant data were reported as the mean ± SD.

## 3. Results and Discussion

### 3.1. Structural Characterization of the Isolated Compound

#### 3.1.1. Melting Point

[270–272°C], [259–271°C, Lit.].

#### 3.1.2. Configuration

Optical rotation: ([*α*]_D_^20^) = −3.0° (c 0.02, ethanol). The configuration at C-2 of the isolated compound (eriodictyol) was deduced as “S” (rotates plane-polarized light counterclockwise, levorotary), thus (S)-2-(3′,4′-dihydroxyphenyl)-5,7-dihydroxychroman-4-one.

#### 3.1.3. HPLC Profile

(Figure, Supplementary Information (SI 1)): the analytical HPLC chromatogram of the isolated compound is indicated in (Table, SI 2, Peak 4). The HPLC chromatogram shows a distinct single compound of high % purity: ((>98%), retention time (*Rt*): 23.95 min), confirming the isolated compound as eriodictyol ((S)-2-(3′,4′-dihydroxyphenyl)-5,7-dihydroxychroman-4-one).

#### 3.1.4. FTIR Spectrum

The FTIR spectrum (Figure, SI 3) indicates the identification of major functional groups such as O-H stretch (3,354 cm^−1^), aliphatic C-H stretch (2,849 cm^−1^, 2,916 cm^−1^), conjugated carbonyl C=O stretch (1,656 cm^−1^), aromatic C=C stretch (1,600 cm^−1^), methylene C-H bend, C-O, C-O-C, C-C-O, C-C stretches, phenolic O-H bend, and ring C-H bend (1,446−700 cm^−1^).

#### 3.1.5. ESI-MS

**Figure 1:**
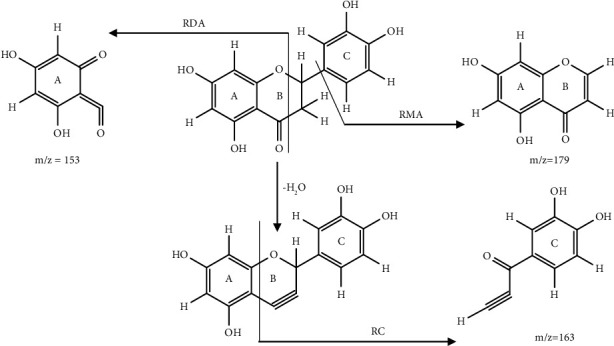
Illustration of the fragmentation products of the isolated compound (eriodictyol).

(Figure, SI 4). (Positive ion mode, 100 V): major ions (m/z): (289.3 [M + H]^+^, 179 [M + H-110]^+^, 163 [M + H-126]^+^, and 153 [M + H-136]^+^). The mechanism underlying the three major fragments in the isolated compound [29–31] involving (a) retro-Diels-Alder (RDA) cleavage with a major fragment having a mass m/z of 153, (b) the retrocyclization (RC) cleavage leading to a major fragment with a mass m/z of 163, and (c) the retro-Michael's addition (RMA) cleavage with a major fragment having a mass m/z of 179 is illustrated in Figure 1.

#### 3.1.6. NMR (Acetone-d6)

**Figure 2:**
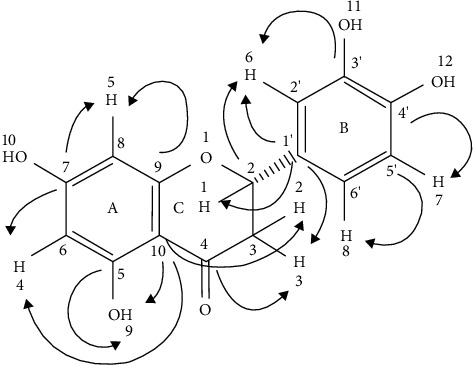
Illustration of HMBC connectivity of rings A, B, and C in the isolated compound.

The summary of the ^1^H (Figure, SI 5), ^13^C (Figure, SI 6), HSQC (Figure, SI 7), and DEPT-135 (Figure, SI 8), illustration of the HMBC connectivity (Figure SI 9, Figure 2), and comparison of the experimental data in the isolated compound with the literature data for eriodictyol are presented in Tables 1 and 2.

In view of the analysis of all the spectral data, the isolated compound was identified as eriodictyol (5,7-dihydroxy-3′,4′-dihydroxyflavanone) and is being reported for the first time from the bark of *A. africana*.

### 3.2. Antimicrobial Activity

The antimicrobial activity of the isolated compound was assessed against 10 different microbes by determining its bacteriostatic and bactericidal effects (MICs, MBCs, and MBC/MIC ratios) over a concentration range of 100.0−0.1 mg/mL. The results of the antimicrobial assay are shown in Table 3 as follows.

According to Mogana et al., compounds with MIC ≤ 2 mg/mL and MIC > 2–8 mg/mL have been found to exhibit some degree of good and weaker inhibitory or bacteriostatic effects, respectively [26]. Based on this classification, the compound was found to possess various degrees of bacteriostatic effects (MIC ≤ 8 mg/mL) against all the microbial strains under consideration. The highest level of inhibitory effects was reported against SM, SA, MRSA, BS, PA, ST, and CA1 (MIC ≤ 2 mg/mL) whilst the weakest (2 mg/mL ≥ MIC ≤ 8 mg/mL) activity was reported against EC, CA2, and KP. Furthermore, the compound exhibited a broad spectrum of bactericidal effects against all the microbial strains at concentrations ranging from 1.5 to 100 mg/mL. Much lower concentrations (MIC ≤ 8 mg/mL) were required for the bactericidal activity of most of the microbial strains, except SA (MBC; 25.0 mg/mL), EC (MBC; 12.50 mg/mL), CA2 (MBC; 100.0 mg/mL), and MRSA (MBC; 25.0 mg/mL). The results suggest that higher concentrations of the compound are required to kill those microbial strains completely. The bactericidal activities were further corroborated by the MBC/MIC ratios where MBC/MIC ≤ 4 were classified as bactericidal agents [26, 34–37]. For instance, the compound (eriodictyol) exhibited bactericidal activities (MBC/MIC ≤ 4) against SM, EC, BS, KP, PA, ST, and fungicidal activity against CA2 (standard strain). The compound also showed good bacteriostatic activities (MBC/MIC > 4.0) against SA, methicillin-resistant SA, and fungistatic activity against fluconazole-resistant CA2 (clinical strain).

The antimicrobial results further confirm similar experiments reported for various extracts of the plant as well as the isolated compound. For instance, the leaves, barks, roots, and seeds of *A. africana* have registered a broad spectrum of biological activities such as antimicrobial, antiplasmodial, antitrypanocidal, antioxidant, anti-inflammatory, and analgesic properties [9–12, 38]. Specific authentication of the antimicrobial activities of *A. africana* was confirmed for the methanol extract of the bark with higher susceptibility for *Staphylococcus aureus* and *Bacillus subtilis.* The extract was however reported to be inactive against *Pseudomonas aeruginosa, Escherichia coli,* and *Salmonella typhi* [10]. Agbelusi et al. investigated the synergistic effects of chewing stick extracts with standard antibiotics which were confirmed against the drug-resistant strain of some bacterial isolates [11]. Moreover, the isolate was also reported to exhibit antimicrobial activities against SA, CA, and EC [39, 40]. The series of reported activities further corroborate those reported for the compound in this study. The antimicrobial activities of the isolated compound and its flavonoid classes have been associated with the presence of *α*-*β* unsaturated carbonyl and multiple phenolic groups in their structures. The structural motifs in these compounds are known to enhance their antimicrobial actions by either interfering or inhibiting bacterial cell wall and protein synthesis at the 30S subunit of ribosomes. In fact, the motifs in these phytochemicals are known to enhance bacterial cell wall permeability for the expression of their antimicrobial activities [41, 42]. Furthermore, flavonoids exhibit their antibacterial action by causing cell lyses of pathogenic bacteria [40, 41]. The isolated compound (eriodictyol) and its flavonoid families such as naringenin have been identified as inhibitors of *β*-ketoacyl acyl carrier protein synthase III (KAS III), a key protein involved in the initiation of fatty acid synthesis in bacterial. The utilization of the compound would therefore serve as a key approach to overcoming antibiotic drug resistance associated with the methicillin-resistant bacteria *Staphylococcus aureus* and other antibacterial resistance [41, 43].

#### 3.2.1. Antibacterial Synergistic Activity

In view of the series of bactericidal activities reported by the compound, further tests to determine its synergistic activity with standard antibacterial agents such as ciprofloxacin and tetracycline were performed (Table 4).

The data suggest that the compound reported synergistic activity against EC, KP, ST, and MRSA; partial synergism against SA; antagonism against PA; and no synergistic effect (indifferent) against SM and BS when combined with ciprofloxacin. The compound further showed synergistic activity with tetracycline against ST, and partial synergism against EC, KP, SA, SM, MRSA, BS, and PA. The mechanistic account for the reported synergistic effects could be linked to its flavanone phytochemical class, and its accompanying structural motifs. The phytochemical class is known to exhibit synergistic effects with several antibiotic agents against several bacterial strains [44, 45]. For instance, their combination with ciprofloxacin and tetracyclines is known to exhibit synergistic effects against *S. aureus*, its methicillin-resistant strains, and several other bacterial strains [44, 45]. The results, therefore, corroborate the synergistic effects reported by the compound against *S. aureus*, methicillin-resistant* S. aureus,* and the other microbial strains in this study. They exhibit their synergistic effects by facilitating the interaction of the antibiotic with its target site in the pathogen. The mechanisms of action have been established to proceed through the major facilitator superfamily (MSF) and the multidrug resistant (MDR) efflux pump pathway. They are also known to enhance the activities of antibiotic interaction by disintegrating the oily outer membrane of bacteria, leading to membrane permeability for the expression of antibiotic activity [44–46].

#### 3.2.2. Antifungal Synergistic Activity

The antifungal synergistic activity of the isolated compound when combined with fluconazole, ketoconazole, and nystatin was determined with the checkerboard assay (Table 5). The interpretation of the synergistic activity was based on the criteria adopted by Yang et al. where the interaction was considered synergistic (S) if the FIC index was ≤0.5; partial synergistic (PS) if the FIC index was >0.5 and <1; additive (AD) if the FIC index was = 1; no difference (indifferent, (I)) if the FIC index was >1 and ≤4; and antagonistic (A) if the FIC index was >4.0 [37, 38]. The study revealed the isolated compound (eriodictyol) reporting synergistic and partial activity against CA2 and CA1, respectively, when combined with fluconazole. Moreover, when the compound was combined with ketoconazole, antagonistic, and additive were reported against CA1 and CA2, respectively. Finally, synergistic and indifferent activity were reported against CA2 and CA1, respectively, when combined with nystatin.

The myriad of synergistic activities reported for the compound further re-enforces the vast antimicrobial activities reported for *A. africana* against *C. albicans* strains. The identified compound and its flavonoid derivatives had been reported against *C. albicans*. They exhibited their synergistic effects by decreasing the ability of cells to efflux out the antibiotics [47]. The effects lead to the permeability, bioavailability, and effectiveness of antibiotics at their target sites [47]. The flavonoid compounds were also known to perform their synergistic effects by inhibiting cell-cell adhesion communication in the fungal strain, leading to the disruption of gene expression responsible for biofilm formation in the fungal strain [48].

### 3.3. Antioxidant Activity

The antioxidant activity of the compound was determined against the ABTS- and DPPH-free radicals scavenging with a concentration range of (100.00−3.13 *µ*g/mL). The study shows significant free radical scavenging activity for the isolated compound against the ABTS and DPPH radicals with SC_50_ (*µ*g/mL) values and ascorbic acid indicated in Table 6 and Figure, SI 10.

The study further reveals the isolated compound indicating superior activity against standard ascorbic acid. The compound belonging to the flavonoid and phenolic phytochemical class further confirms the vast antioxidant activities reported for such a class of compounds [49]. The antioxidant activities of *A. africana* and its fractions had been reported for various parts of the plant but no compound was assigned to their reported activities [38]. The isolated compound, therefore, gives further credence to the utilization of *A. africana* in managing medical conditions related to oxidative stress. The antioxidant activities of the identified compound and its flavonoid derivatives have been linked to the presence of multiple phenolic groups in their structures. The phenolic groups in their structures perform their antioxidant activities by acting as pro-oxidants by chelating metals and other reactive radicals [49]. Such phytochemicals are good hydrogen donors and normally react with reactive oxygen and nitrogen radicals. The resultant effect could terminate the generation of free radicals and consequently break the cycle of the generation of new ones [26, 50]. Several mechanistic pathways have been reported for the numerous medicinal potentials of the compound (eriodictyol). In fact, the compound had been reported to protect the LPS-triggered oxidative stress, neuroinflammation, and synaptic dysfunction through the MAPK NF‐*κ*B mediated by ROS, Sirt1, and Nrf2/Keap1 signal pathways. The reported property confirms the antioxidant activity reported in this study and could be applied as a nutraceutical for managing oxidative-related neurodegenerative ailments and diseases [18]. Furthermore, the Fc″RI-mediated human basophilic KU812F cells antiallergic activity of the 7-O-*β*-d-glucuronide derivative of the compound (eriodictyol) and its chromene moiety, 5,7-dihydroxy-4-chromene have been investigated. The study concluded that the compounds can downregulate protein and messenger RNA (mRNA) expression of Fc″RI on the cell surface implicating its potential for the treatment of allergic disorders [51]. In other studies, eriodictyol was identified as a novel insulin secretagogue through the stimulation of the cAMP/PKA signaling pathway at higher glucose concentration and hence could be applied as prophylaxis for treating drug-induced hypoglycemia [52]. Moreover, the anti-inflammatory, antioxidant, antimicrobial, immunomodulatory, cytoprotective effects on the kidney, hepatoprotective, neuroprotective, analgesic, antipyretic, anticancer, and antiallergenic were well documented [41, 51–57]. These numerous pharmacological activities reported for the plant could contribute to the vast medicinal potentials reported for *A. africana*.

## 4. Conclusion

A flavanone derivative, eriodictyol ((S)-2-(3ʹ,4ʹ-dihydroxyphenyl)-5,7-dihydroxychroman-4-one), was isolated for the first time from the bark of *A. africana*. The compound exhibited significant antioxidant activity against ABTS and DPPH radicals and a broad spectrum of antimicrobial and synergistic effects against several bacterial and fungal strains. The study gives scientific credence to the numerous ethnobotanical and medicinal properties of *A. africana*.

## Figures and Tables

**Table 1:** Summary of the spectral data for the isolated compound (eriodictyol).

Carbons	^13^C-NMR	DEPT-135	^1^H-NMR	HSQC	*HMBC*		
					^1^H	^2^J	^3^J
C2	80.0	CH	5.39 (H1)	C2-H1	H1	H1-C1′	H1-C2′, H1-C6′
C3	43.6	CH_2_	2.70 (H2)	C3-H2	H2	H2-C4	H2-C10
			3.11 (H3)	C3-H3	H3	H3-C2, H3-C4	H3-C1′
C4	197.2	—	—	—		H2-C4, H3-C4	—
C5	165.3	—	12.17 (H9, OH)	—	H9	H9-C5	H9-C6, H9-C10
C6	96.7	CH	5.94 (H4)	C6-H4	H4	H4-C5, H4-C7	H4-C8, H4-C10
C7	167.4	—	8.50 (H10, OH)	—	H10	H5-C7, H4-C7	—
C8	95.8	CH	5.95 (H5)	C8-H5	H5	H5-C9, H5-C7	H5-C6
C9	164.3	—	—	—		H5-C9	—
C10	103.2	—	—	—		—	H4-C10, H9-C10
C1′	131.5	—	—	—		H1-C1′	H3-C1′, H7-C1′
C2′	114.7	CH	7.03 (H6)	C2′-H6	H6	H6-C3′	H6-C2, H6-C6′
C3′	146.0	—	8.50 (H11, OH)	—	H11	H6-C3′	—
C4′	146.4	—	8.50 (H12, OH)	—	H12	H7-C4′	—
C5′	116.0	CH	6.87 (H7)	C5′-H7	H7	H7-C4′	—
C6′	119.2	CH	6.87 (H8)	C6′-H8	H8	H8-C1′, H8-C5′	H8-C2, H8-C2′

**Table 2:** Comparison of spectral data of the isolated compound with literature.

Mass spectrometry fragmentation (m/z)		Carbon number	NMR spectral data (ppm)						IR peaks (cm^−1^)	
			^13^C-NMR peaks			^1^H-NMR peaks				
Isolate MeOH	Lit. MeOH [32]		Isolate Acetone-d6	Lit. MeOD [33]	Lit. (DMSO-d6) [32]	Isolate Acetone-d6	Lit. MeOD [33]	Lit. (DMSO-d6) [32]	Isolate Neat	Lit. MeOH [32]
153.0	153.0	C2	80.0	80.5	78.5	5.39	5.27	5.36	3354	3367
163.0	163.0	C3	43.6	44.1	42.1	2.70	2.69	2.66	2916	—
179.0	179.0	C4	197.2	197.7	196.3	3.11	3.06	3.15	2849	—
288.3	288.0	C5	165.3	165.4	163.6	12.17	—	12.15	1656	1636
		C6	96.7	97.0	95.8	5.94	5.86	5.85	1599	1602
Melting point (°C)		C7	167.4	168.4	166.6	8.50	—	9.05	1449	1447
[270–272°C]		C8	95.8	96.1	95.0	5.95	5.88	5.85	1157	1160
[259–271°C]: lit. [32]		C9	164.3	164.8	163.5	—	—	—	1082	1086
		C10	103.2	103.3	101.8	—	—	—		
		C1′	131.5	131.7	129.5	—	—	—		
		C2′	114.7	114.6	114.4	7.03	6.90	6.87		
		C3′	146.0	146.5	145.2	8.50	—	9.05		
		C4′	146.4	146.9	145.7	8.50	—	9.05		
		C5′	116.0	116.2	115.4	6.87	6.77	6.74		
		C6′	119.2	119.2	117.9	6.87	6.78	6.74		

**Table 3:** MBCs, MICs, and MBC/MIC ratios for the isolated compound and antibacterial agents.

Bacterial strains	*Antibacterial activity of isolate (MIC, MBC) (mg/mL)*			*MICs of standard antibacterial agent (µg/mL)*	
	MIC	MBC	MBC/MIC ratio	CIPRO	TET
SM	0.39	1.56	4.00^bc^	125.00	7.81
SA	1.56	25.00	16.00^bs^	7.81	7.81
EC	3.13	12.50	4.00^bc^	125.00	7.81
CA2	6.25	100.00	16.00^bs^	125.00	7.81
MRSA	0.20	25.00	125.00^bs^	250.00	15.63
BS	1.56	3.13	2.00^bc^	125.00	7.81
KP	3.13	6.25	2.00^bc^	125.00	7.81
PA	0.39	1.56	4.00^bc^	125.00	7.81
ST	1.56	1.56	1.00^bc^	3.90	15.63
CA1	0.78	3.13	4.00^bc^	—	—

^bc^Bactericidal activity; ^bs^Bacteriostatic activity. CIPRO: ciprofloxacin, TET: tetracycline, SM: *Streptococcus mutans*, SA: *Staphylococcus aureus*, EC: *Escherichia coli*, CA2: fluconazole-resistant *Candida albicans* 2 (clinical strain), MRSA: methicillin-resistant *Staphylococcus aureus*, BS: *Bacillus subtilis*, KP: *Klebsiella pneumonia*, PA: *Pseudomonas aeruginosa*, ST: *Salmonella typhi*, and CA1: *Candida albicans* 1 (standard strain).

**Table 4:** Synergistic activity of the isolated compound with antibacterial agents against bacterial strains.

Strains	MIC (CIPRO) (*µ*g/mL)	FIC (CIPRO + isolate)	INT	MIC (TET)	FIC (TET + isolate)	INT
EC	125.00	0.38	S	7.81	0.56	PS
KP	125.00	0.38	S	7.81	0.56	PS
SA	7.81	0.63	PS	7.81	0.57	PS
SM	125.00	3.71	I	7.81	0.62	PS
ST	3.90	0.01	S	15.63	0.31	S
MRSA	250.00	0.38	S	7.81	0.57	PS
BS	125.00	1.50	I	7.81	0.51	PS
PA	125.00	5.00	A	7.81	0.51	PS

FIC: fractional inhibitory concentration, CIPRO: ciprofloxacin, and TET: tetracycline. INT: interpretation (synergistic (S) if the FIC index ≤ 0.5; partial synergistic (PS) if the FIC index >0.5 and <1; additive (AD) if the FIC index = 1; no synergism or indifferent (I) if the FIC index >1 and ≤4; antagonistic (A) if the FIC index >4.0).

**Table 5:** Synergistic activity of compound with antifungal agents against fungal strains.

Agents	*CA1*	*CA2*	Combination	*CA1*	INT	*CA2*	INT
	MIC	MIC		FIC index		FIC index	
FLC	64	>64	Isolate + FLC	0.563	PS	0.312	S
KET	512	512	Isolate + KET	4.063	A	1.000	AD
NYX	128	128	Isolate + NYS	1.319	I	0.356	S

FLC: fluconazole; KET: ketoconazole; NYX: nystatin; INT: interpretation.

**Table 6:** SC_50_ values for eriodictyol and ascorbic acid.

Samples	Tests	Regression equations	SC_50_ (*µ*g/mL) = log^−1^[*x*] @ *y* = 50
Eriodictyol	ABTS	*y* = 29.32*x* + 40.37	2.14 ± 0.05
	DPPH	*y* = 33.10*x* + 36.82	2.51 ± 0.06

Ascorbic acid	ABTS	*y* = 35.40*x* + 25.33	5.00 ± 0.12
	DPPH	*y* = 45.07*x* + 10.41	7.58 ± 0.12

## Data Availability

All the data used to support the findings of the study are included within the article.

## Supplementary Materials

Supplementary Materials The supplementary document consists of the HPLC, FTIR, MS, ^1^H, ^13^C, HSQC, and DEPT-135 NMR as well as the HMBC connectivity and the antioxidant data results. This is presented in Supplementary Information (SI) File in the following. SI 1: HPLC chromatogram of the isolated compound. SI 2: peak area and retention time for the isolated compound and minor impurities. SI 3: FTIR spectrum of the isolated compound. SI 4: mass spectrum (MS) of the isolated compound. SI 5: ^1^H-NMR spectrum of the isolated compound. SI 6: ^13^C-NMR spectrum of the isolated compound. SI 7: HSQC-NMR spectrum of the isolated compound. SI 8: DEPT-135 NMR spectrum of the isolated compound. SI 9: illustration of HMBC connectivity in the isolated compound. SI 10: a graph of % ABTS and DPPH scavenging activity versus Log [Concentration (*µ*g/mL)] for the isolated compound.
