# Supplementary material for: Isolation and Identification of Flavanone Derivative Eriodictyol from the Methanol Extract of Afzelia africana Bark and Its Antimicrobial and Antioxidant Activities
Source: Evid Based Complement Alternat Med. 2023 May 9;2023:9345047. doi: 10.1155/2023/9345047 (PMC10188263; doi:10.1155/2023/9345047)
Supplement: Supplementary Materials — The supplementary document consists of the HPLC, FTIR, MS, 1H, 13C, HSQC, and DEPT-135 NMR as well as the HMBC connectivity and the antioxidant data results. This is presented in Supplementary Information (SI) File in the following. SI 1: HPLC chromatogram of the isolated compound. SI 2: peak area and retention time for the isolated compound and minor impurities. SI 3: FTIR spectrum of the isolated compound. SI 4: mass spectrum (MS) of the isolated compound. SI 5: 1H-NMR spectrum of the isolated compound. SI 6: 13C-NMR spectrum of the isolated compound. SI 7: HSQC-NMR spectrum of the isolated compound. SI 8: DEPT-135 NMR spectrum of the isolated compound. SI 9: illustration of HMBC connectivity in the isolated compound. SI 10: a graph of % ABTS and DPPH scavenging activity versus Log [Concentration (µg/mL)] for the isolated compound. [file 9345047.f1.docx]

**Isolation and identification of flavanone derivative, eriodictyol from the methanol extract of *Afzelia africana* bark and its antimicrobial and antioxidant activities**

Bright Yaw Vigbedor,^1^ Clement Osei Akoto,^2^* ^1^David Neglo^1^

^1^Department of Basic Sciences, School of Basic and Biomedical Sciences, University of Health and Allied Sciences, Ho, Ghana.

^2^Department of Chemistry, Faculty of Physical and Computational Sciences, College of Science, Kwame Nkrumah University of Science and Technology (KNUST).

*Correspondence should be addressed to:

Clement Osei Akoto; [cakoto@hotmail.com](mailto:cakoto@hotmail.com), [cakoto@ualberta.net](mailto:cakoto@ualberta.net).

Clement Osei Akoto

Email: [cakoto@hotmail.com](mailto:cakoto@hotmail.com) / [oseiakotoclement@knust.edu.gh](mailto:oseiakotoclement@knust.edu.gh) / [cakoto@ualberta.net](file:///C:\Users\CLEMENT\Documents\PROJECT-KNUST-2018-2019\MANUSCRIPT\cakoto@ualberta.net)

ORCID: 0000 0003 2717 2753

Bright Yaw Vigbedor

Email: [vigbedorphd@yahoo.com / ybvigbedor@uahs.edu.gh](mailto:vigbedorphd@yahoo.com%20/%20ybvigbedor@uahs.edu.gh)

ORCID: 0000 0003 3955 9883

David Neglo

Email: dneglo@uhas.edu.gh

ORCID: 0000 0003 2547 5021

SUPPORTING INFORMATION (SI)

S1 1: HPLC chromatogram of the isolated compound

SI 2: Peak area and retention time for the isolated compound and minor impurities

| Peak | Retention time/min | Area | Percentage of component (%) |
| --- | --- | --- | --- |
| 1 | 3.08 | 2,097.47 | 0.06 |
| 2 | 14.5 | 3,145.21 | 0.09 |
| 3 | 20.92 | 30443.3 | 0.88 |
| 4 | 23.95 | 3,391,934.40 | 98.29 |
| 5 | 25.50 | 20,447.50 | 0.59 |
| 6 | 34.10 | 3,047.56 | 0.09 |
| Total | - | 3,451,115.44 | 100.00 |

S1 3: FTIR spectrum of the isolated compound


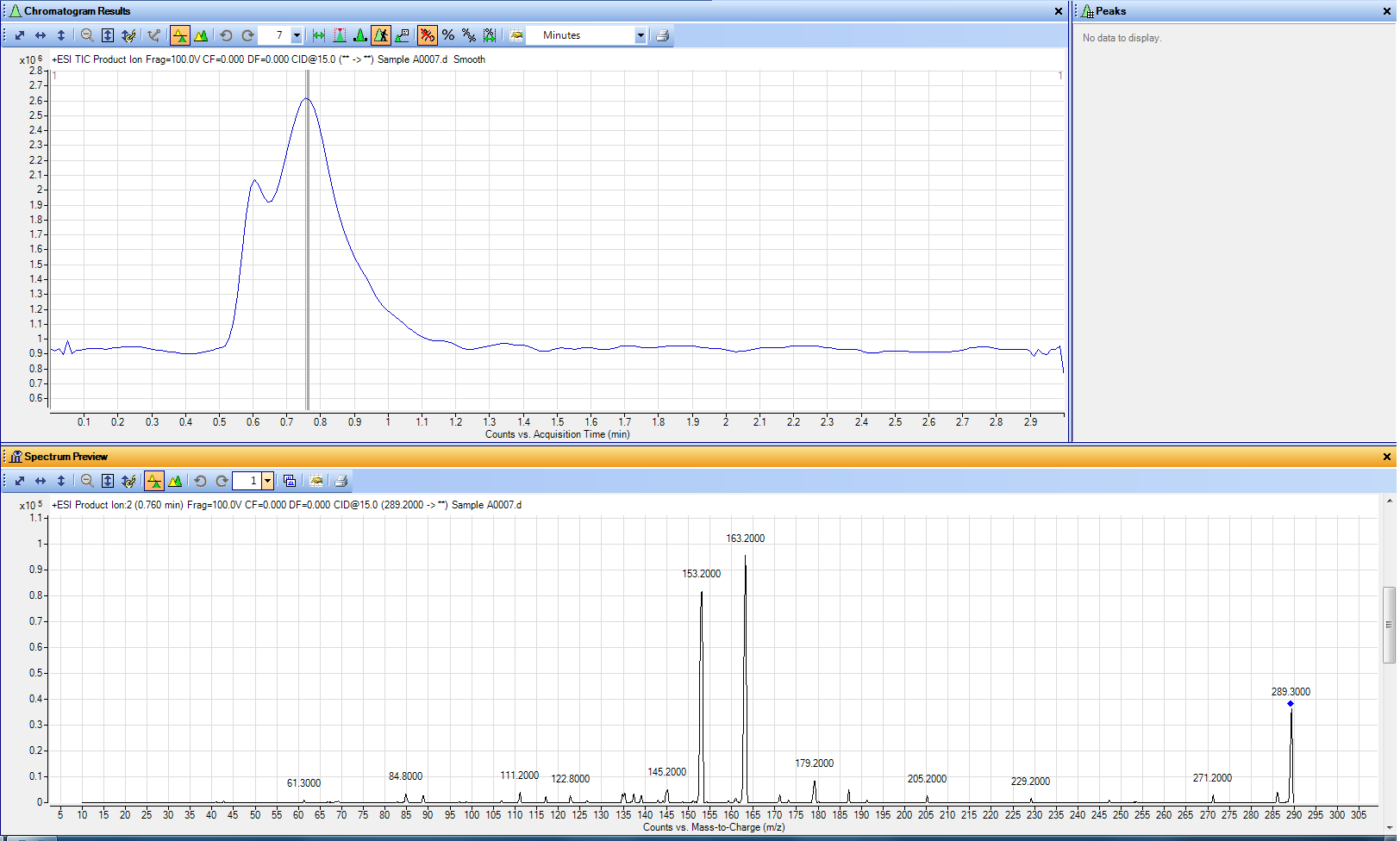

SI 4: Mass spectrum (MS) of the isolated compound

H1

H3

H5

H9

[H7, H8]

[H12, H10, H11]

H_2_O

Acetone-d6

H6

H4

H2

SI 5: ^1^H - NMR spectrum of the isolated compound

Acetone-d6

C3

C2

C6

C8

C1'

C6'

C3'

C4'

C2'

C5'

C5

C9

**

C7

C10

C4

SI 6: ^13^C - NMR spectrum of the isolated compound

H8

H7

Acetone-d6

H_2_O

[C2'-H6](114.7, 7.03)

[C3-H2](43.6, 2.70)

[C2-H1](80.0, 5.39)

[C6'-H8](119.2, 6.87)

[C5'-H7](116.0, 6.87)

[C6-H4](96.7, 5.94)]

[C8-H5](95.8, 5.95)

[C3-H3] (43.6, 3.11)]


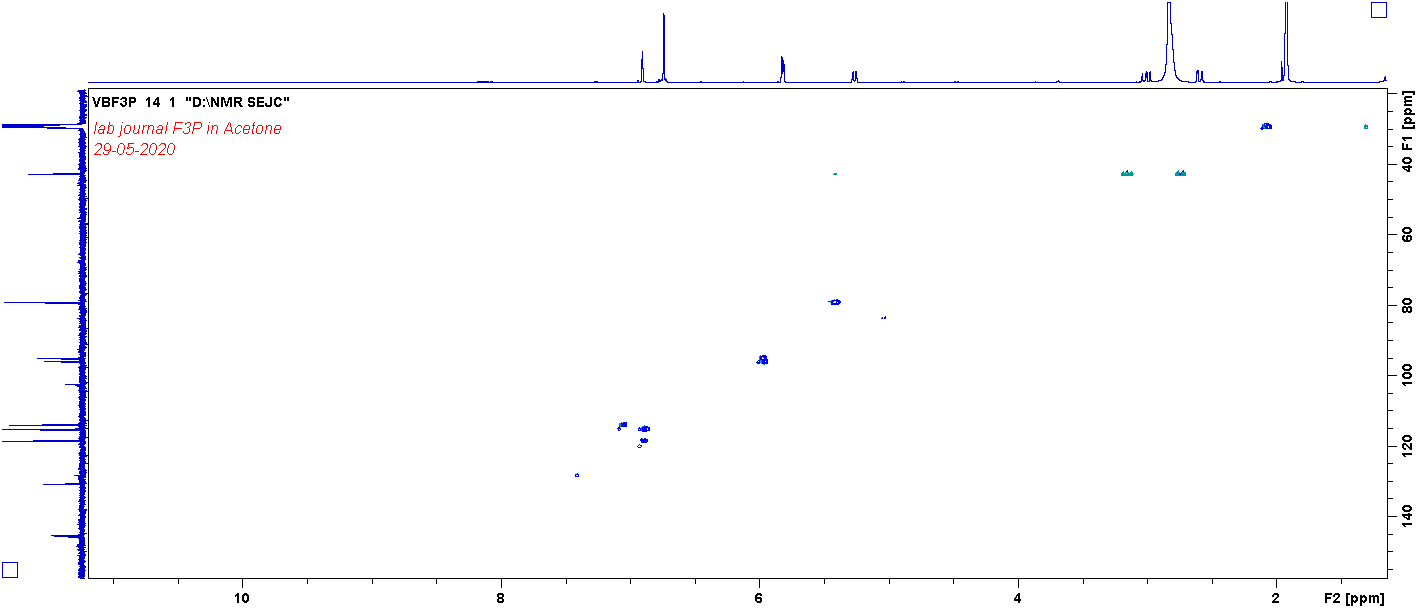


C3

C2

C2'

C6'

C5'

C6

C8

H4

H5

H2

H3

H1

H6

SI 7: HSQC-NMR spectrum of the isolated compound

Acetone-d6

C5'(CH:116.0)

**
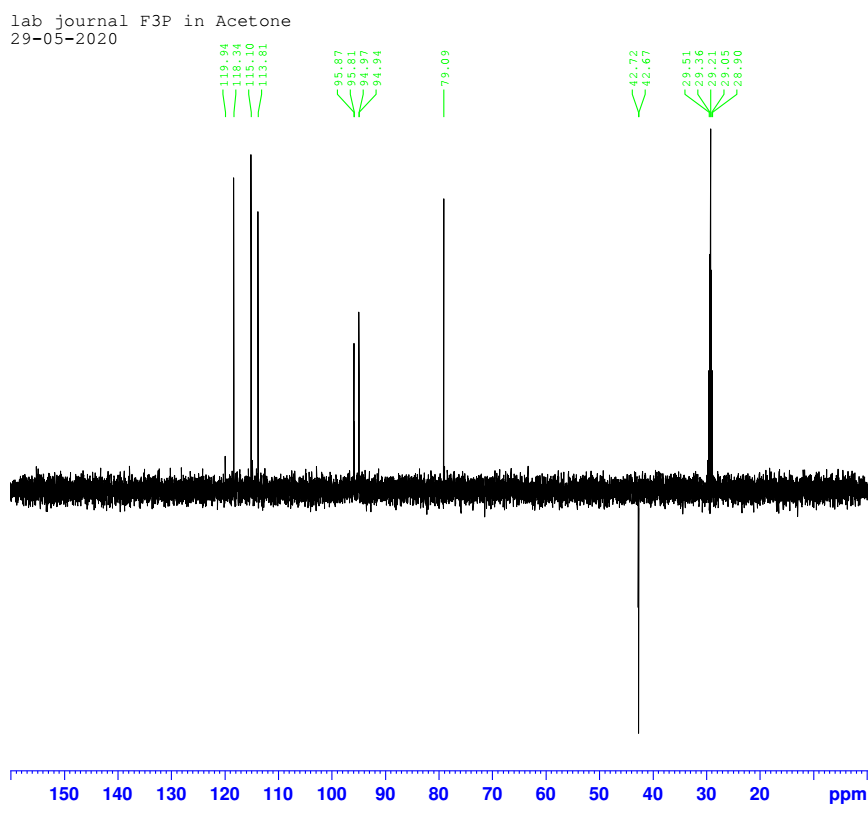
**

C3(CH_2_: 43.6)

C6(CH: 96.7)

C2(CH: 80.0)

C8(CH: 95.8)

C6'(CH:119.2)

C2'(CH: 114.7)

SI 8: DEPT-135 NMR spectrum of the isolated

Acetone-d6

H_2_O

C7

C5

[H10 H11 H12]

H8 H7

H5 H4

H6

C6'

C10

C8

C6

C2

C4

[b6]

[a3]

^2^J[C1'-H1]

^3^J[C2'-H1]

^3^J[C6'-H1]

^3^J[C2-H6]

^3^J[C2-H8]

^2^J[C4-H3]

]

^3^J[C1'-H3]

[b1]

[b4]

[b3]

C9

[a8]

[a7]

[a4]

[a5]

[a2]

[a1]

^3^J[C10-H2]

]

^2^J[C2-H3]

]

^2^J[C4-H2]

]

[a6]

^3^J[C6-H9]

]

^3^J[C10-H9]

]

^2^J[C5-H9]

]

[b8]

[b7]

[b5]

[b2]

H3 H2

H1

H9

C4'

C3'

C1'

C5'

C2'

C3

**a1:** (^2^J[C5-H4])); **a2:** (^2^J[C7-H4]); **a3:** (^2^J[C7-H5])); **a4:** (^3^J[C6-H5]); **a5:** (^3^J[C8-H4]); **a6:** (^2^J[C9-H5]); **a7:** (^3^J[C10-H4]); **a8:** (^3^J[C10-H5]); **b1:** (^2^J[C1'-H6]); **b2:** (^2^J[C1'-H8]); **b3:** (^3^J[C1'-H7]); **b4:** (^3^J[C2'-H8]); **b5:** (^2^J[C3'-H6]); **b6:** (^2^J[C(4'-H7]), **b7:** (^2^J[C5'-H8]); **b8:** (^3^J[C6'-H6]).

SI 9: Illustration of HMBC connectivity in the isolated compound

SI 10: A graph of % scavenging activity versus Log [Concentration (µg/mL)]
